# Bone mineral density in high-level endurance runners: part A—site-specific characteristics

**DOI:** 10.1007/s00421-021-04793-3

**Published:** 2021-09-12

**Authors:** A. J. Herbert, A. G. Williams, S. J. Lockey, R. M. Erskine, C. Sale, P. J. Hennis, S. H. Day, G. K. Stebbings

**Affiliations:** 1 School of Health Sciences, Birmingham City University, Birmingham, UK; 2 Sports Genomics Laboratory, Department of Sport and Exercise Sciences, Manchester Metropolitan University, Manchester, UK; 3 Faculty of Health, Education, Medicine and Social Care, Anglia Ruskin University, Chelmsford, UK; 4 School of Sport and Exercise Science, Liverpool John Moores University, Liverpool, UK; 5 Musculoskeletal Physiology Research Group, Sport, Health and Performance Enhancement Research Centre, School of Science and Technology, Nottingham Trent University, Nottingham, UK; 6 School of Medicine and Clinical Practice, University of Wolverhampton, Wolverhampton, UK; 7 Institute of Sport, Exercise and Health, University College London, London, UK

**Keywords:** Bone mineral density, Elite, Marathon, Mechanical loading, Menstruation

## Abstract

**Purpose:**

Physical activity, particularly mechanical loading that results in high-peak force and is multi-directional in nature, increases bone mineral density (BMD). In athletes such as endurance runners, this association is more complex due to other factors such as low energy availability and menstrual dysfunction. Moreover, many studies of athletes have used small sample sizes and/or athletes of varying abilities, making it difficult to compare BMD phenotypes between studies.

**Method:**

The primary aim of this study was to compare dual-energy X-ray absorptiometry (DXA) derived bone phenotypes of high-level endurance runners (58 women and 45 men) to non-athletes (60 women and 52 men). Our secondary aim was to examine the influence of menstrual irregularities and sporting activity completed during childhood on these bone phenotypes.

**Results:**

Female runners had higher leg (4%) but not total body or lumbar spine BMD than female non-athletes. Male runners had lower lumbar spine (9%) but similar total and leg BMD compared to male non-athletes, suggesting that high levels of site-specific mechanical loading was advantageous for BMD in females only and a potential presence of reduced energy availability in males. Menstrual status in females and the number of sports completed in childhood in males and females had no influence on bone phenotypes within the runners.

**Conclusion:**

Given the large variability in BMD in runners and non-athletes, other factors such as variation in genetic make-up alongside mechanical loading probably influence BMD across the adult lifespan.

**Supplementary Information:**

The online version contains supplementary material available at 10.1007/s00421-021-04793-3.

## Introduction

Bone mineral density (BMD) is considered to be the primary predictor of osteoporotic fracture (Cranney et al. 2007) although other factors such as geometry, architecture and collagen properties are important determinants of bone strength (Cheung et al. 2016). BMD may also be important for success in the elite sporting environment due to the potential influence on training, performance and injury (Herbert et al. 2019). Diet, hormones and genetics are all known to influence BMD and contribute to large variability within the phenotype (Pluijm et al. 2001). In addition, mechanical loading experienced during regular physical activity influences BMD, with load-bearing, high-impact sports associated with increased strain rates, higher peak-force loading and higher BMD (Andreoli et al. 2001). However, the influence of mechanical loading on BMD in endurance athletes is more complex.

Whilst higher leg BMD has been reported in male runners in comparison to non-athletes (Stewart and Hannan 2000; Kemmler et al. 2006) across adulthood (Velez et al. 2008), and in female adolescent runners compared to non-athletes at multiple sites (Duncan et al. 2002), some endurance runners may be at risk of low BMD. In endurance runners, low BMD may increase the risk of stress fracture, which can have negative implications for health and performance (Hind et al. 2006; Barrack et al. 2008; Pollock et al. 2010). Excessive training volumes and/or insufficient dietary intake by endurance runners can result in reduced energy availability, which can negatively impact metabolic processes and potentially reduce BMD (Loucks 2007). Hind et al. (2006) showed low lumbar spine BMD (< − 1.0 T-score) in 37% of 44 male runners aged 19–50 years in comparison with the manufacturer’s reference control database (Lunar Prodigy, GE Systems, UK), whilst lower lumbar spine BMD (Z-score of − 1.0 to − 2.0) has also been reported in adolescent and adult female runners (Barrack et al. 2008; Pollock et al. 2010). Lower BMD was more evident in those runners exhibiting menstrual irregularities (Pollock et al. 2010) or where dietary restraint was occurring (Barrack et al. 2008).

Possession of low or lower BMD by some runners in comparison to other runners may be influenced by a number of factors. Low energy availability and/or menstrual dysfunction, for example, may explain why low or lower BMD is particularly evident in some female runners compared to other female runners or non-athletes (Pollock et al. 2010; Scofield and Hecht 2012). Whilst low energy availability is prevalent in endurance runners, detecting and assessing the impact of menstrual dysfunction and/or low energy availability on BMD and other phenotypes is difficult (Heikura et al. 2018).

No studies, to our knowledge, have assessed the impact of sporting activity in childhood on BMD in adulthood in high-level endurance runners. Physical activity during childhood can play an important role in the attainment of peak BMD in adulthood (Tveit et al. 2013; Weaver et al. 2016), but it is difficult to complete studies that are both longitudinal and valid due to the challenge of obtaining accurate measurement of activity. Thus, the impact of childhood physical activity in populations, such as endurance runners, and the outcome for BMD is still unclear (Herbert et al. 2019).

In comparison to non-athletes, runners typically demonstrate higher site-specific BMD but similar, or lower, total body and non-loading site BMD (e.g. lumbar spine), due to the associated mechanical loading on the lower extremity (Scofield and Hecht 2012). It is difficult, however, to directly compare and draw conclusions between the majority of studies in this area due to the substantial differences in methodological design. For example, many studies report on varying sample sizes that comprise athletes of differing ability, which is likely to increase the inter-individual variability within the measured phenotype. Some studies do consist of athletes of a similar ability/standard by recruiting only ‘national’ or ‘regional’ level athletes, but the definitions of what constitutes national or regional level athletes are not always clear and consistent (Swann et al. 2015). BMD comparisons between these populations is, therefore, difficult due to a probable difference in training load characteristics. Furthermore, most of these studies have been conducted on non-elite athletes (i.e. defined here as those who have not competed at international or national level), with only one study thus far comprising wholly UK elite endurance runners (Pollock et al. 2010).

In sports, where ability/success is based upon time to complete a specific distance, such as endurance running, criteria based upon personal best time (PB) rather than representative level arguably allows for better assessment of athlete calibre, because national representation requires faster running in some countries than others. Utilising a large cohort of high-level endurance runners of similar competitive standard (based on PB), many undertaking similar training volumes and/or intensities, may somewhat alleviate the aforementioned issues arising from loosely defined populations and their potential confounding variables.

The primary aim of this investigation was to compare total body (_T_BMD), leg (_L_BMD) lumbar spine BMD (_LS_BMD), and total-body T-score and Z-score between high-level endurance runners (selected based upon PB) and non-athletes. The secondary aims were to assess the influence of menstrual irregularities and sporting activity during childhood on these bone phenotypes.

## Materials and methods

### Participants and participant recruitment

All experimental procedures were conducted in accordance with the guidelines in the Declaration of Helsinki and were approved by the local Ethics Committee of Manchester Metropolitan University. Participants consisted of 103 high-level Caucasian runners (45 males, 58 females) who competed in events ranging from 3000 m to marathon distance and 112 ethnically matched individuals (52 males, 60 females), who did not compete in any sport with a major physical fitness component at regional, national or international level, defined as non-athletes. A sub-group of these participants and some of the associated information and protocols have been described previously when assessing the impact of body composition on stress fracture incidence (Varley et al. 2021). Specifically, runners were primarily recruited from the London Marathon Expo between 2012 and 2015 as well as national/regional athletic clubs and organisations. The non-athlete group were recruited through mail-outs, posters and word of mouth. Race PB time was verified by official race chip timings through individual race result websites, the power of 10 (http://www.thepowerof10.info/) and/or the International Association of Athletics Federations (IAAF) (https://www.iaaf.org/home). Runners were included if they had completed at least one official distance event ≥ 3000 m in a time faster than a predetermined threshold (Table 1). The predetermined threshold time for each distance was chosen to ensure all runners placed in at least the top 600 in the UK rankings for a calendar year based on the years 2012–2017. Average weekly running distance ranged from 24 to 175 km and training hours per week ranged from 8 to 18 h.

**Table 1:** Personal best selection criteria for both male and female runners

Distances	Males	Females
3000 m	< 8 min 45 s	< 10 min 15 s
5000 m/5 km road	< 15 min 45 s	< 18 min 45 s
10,000 m/10 km road	< 32 min 45 s	< 38 min 45 s
Half marathon	< 74 min 00 s	< 88 min 00 s
Marathon	< 2 h 45 min 00 s	< 3 h 15 min 00 s

### Protocol

All runners completed a questionnaire detailing geographic ancestry, performance, training practices and injury, as well as sporting history via an adapted version of the Bone Physical Activity Questionnaire (BPAQ) (Weeks and Beck 2008). This allowed initial assessment of running competition standard and assessed the type and number of sporting activities undertaken in childhood. A sporting activity was included if this had been completed for a minimum of 1 year at least twice per week. To assess the influence of sporting history in childhood on bone phenotypes, the past BPAQ (pBPAQ) algorithm was utilised, whereby the effective load stimulus (derived from previous ground reaction force testing) was multiplied against the number of years of participation and the age weighting factor as developed by Weeks and Beck (2008). One male participant was removed from pBPAQ analysis due to discrepancies in the information provided. Female runners also completed a questionnaire detailing menstruation history that allowed identification of those who demonstrate, or have demonstrated, amenorrheic characteristics. Absence of menses until the age of 16 years or 6 months without menstruation were considered potentially amenorrheic (Gordon and Nelson 2003). Non-athletes completed a questionnaire detailing geographic ancestry, general health and physical activity to establish matched geographic ancestry and ensure no history of high-level sport.

All participants completed a whole-body DXA scan (Hologic Discovery W, Vertec Scientific Ltd, UK) to gather BMD (g/cm^2^) data by one trained operator following the manufacturer’s guidelines. Whole-body and segmental analysis was utilised to obtain _T_BMD, _L_BMD and _LS_BMD. Total-body T-score and total-body Z-score were also acquired via the DXA scan and subsequent analysis. Precision of regional analysis for this DXA model has been reported as 1.1% previously (Ward et al. 2007).

### Statistical analysis

Multiple analysis of variance (MANOVA) was used to compare bone phenotypes (_T_BMD, _L_BMD, _LS_BMD, T-score and Z-score) between the female runners and non-athletes and as well as between male runners and their non-athlete counterparts. Coefficient of variation (CV) for _T_BMD, _L_BMD and _LS_BMD for both male and female runners as well as their non-athlete counterparts was calculated to assess variability. To account for menstruation, the bone phenotypes of female runners who exhibited signs of amenorrhoea were compared with those who were classed as eumenorrheic via MANOVA. Independent *T* tests compared age, height and body mass between runners and non-athletes, whilst body mass-adjusted bone phenotype values were analysed via multiple analysis of covariance (MANCOVA). Linear regression was utilised to investigate whether the calculated pBPAQ score was related to bone phenotypes (_T_BMD, _L_BMD and _LS_BMD) in adulthood. Alpha was set at 0.05 and data are reported as mean (standard deviation) unless otherwise stated.

## Results

Variability (> 16%) for the three BMD site measures existed for both males and females within runners and non-athletes. Differences in body mass (*P* ≤ 0.001) but not age or height (*P* ≤ 0.760) were present between runners and non-athletes for both males and females (Tables 2 and 3). A significant difference in bone phenotypes between runners and non-athletes was shown for both males and females when adjusted and not adjusted for body mass (*P* ≤ 0.004).

**Table 2:** Anthropometric characteristics and bone phenotype data in male high-level endurance runners and non-athletes

	Runners (*n* = 45)	Non-athletes (*n* = 52)	*P* value	95% CI of the mean difference
Age (years)	36 (9)	35 (14)	0.565	− 3.360 to 6.118
Height (m)	1.78 (0.06)	1.79 (0.07)	0.354	− 0.034 to 0.018
Mass (kg)	66.9 (6.6)	78.0 (10.8)	< 0.001	− 14.770 to − 7.438
Fat mass (kg)	11.4 (4.3)	17.6 (6.5)	< 0.001	− 8.463 to − 4.038
Lean mass (kg)	52.8 (4.97)	57.3 (6.5)	< 0.001	− 6.776 to − 2.124
_T_BMD (g/cm^2^)	1.285 (0.094)	1.315 (0.114)	0.176	− 0.072 to 0.013
CV (%)	7.30	8.71	N/A	N/A
Adj _T_BMD (g/cm^2^)	1.325 (0.014)*	1.281 (0.013)	0.036	0.003–0.086
_L_BMD (g/cm^2^)	1.477 (0.108)	1.476 (0.132)	0.963	− 0.048 to 0.050
CV (%)	7.33	8.93	N/A	N/A
Adj _L_BMD (g/cm^2^)	1.523 (0.016)*	1.436 (0.015)	< 0.001	0.040–0.135
_LS_BMD (g/cm^2^)	1.088 (0.151)*	1.189 (0.181)	0.004	− 0.170 to − 0.034
CV (%)	13.84	15.25	N/A	N/A
Adj _LS_BMD (g/cm^2^)	1.123 (0.026)	1.159 (0.024)	0.345	− 0.112 to 0.040
T-score	0.84 (0.88)	1.14 (1.07)	0.141	− 0.697 to 0.100
Adj T-score	1.19 (0.14)	0.84 (0.13)	0.091	− 0.055 to 0.742
Z-score	0.82 (0.85)	1.13 (0.95)	0.092	− 0.678 to 0.052
Adj Z-score	1.14 (0.12)	0.85 (0.11)	0.106	− 0.064 to 0.656
_T_BMD range	0.488 (1.034–1.522)	0.564 (1.067–1.631)	N/A	N/A
_L_BMD range	0.526 (1.193–1.719)	0.749 (1.036–1.785)	N/A	N/A
_LS_BMD range	0.812 (0.750–1.560)	0.818 (0.830–1.650)	N/A	N/A
T-score range	4.60 (− 1.70–2.90)	5.10 (− 1.40–3.70)	N/A	N/A
Z-score range	4.30 (− 1.60–2.70)	4.70 (− 0.90–3.80)	N/A	N/A

Data are mean (SD) except adjusted values that are mean (SE), range variables that are mean (minimum–maximum) and coefficients of variation (CV) that are percentages

_*T*_*BMD* total bone mineral density, _*L*_*BMD* leg bone mineral density, _*LS*_*BMD* lumbar spine bone mineral density, *Adj* adjusted

*Indicates difference from non-athletes

**Table 3:** Anthropometric characteristics and bone phenotype data in female high-level endurance runners and non-athletes

	Runners (*n* = 58)	Non-athletes (*n* = 60)	*P* value	95% CI of the mean difference
Age (years)	34 (12)	38 (16)	0.235	− 8.287 to 2.053
Height (m)	1.65 (0.06)	1.64 (0.06)	0.760	− 0.019 to 0.025
Mass (kg)	52.9 (5.2)	64.7 (11.4)	< 0.001	− 14.970 to − 8.480
Fat mass (kg)	12.2 (3.2)	22.7 (8.1)	< 0.001	− 12.699 to − 8.186
Lean mass (kg)	38.7 (3.6)	39.2 (5.9)	0.615	− 2.225 to 1.322
_T_BMD (g/cm^2^)	1.203 (0.088)	1.191 (0.108)	0.508	− 0.024 to 0.480
CV (%)	7.32	9.09	N/A	N/A
Adj _T_BMD (g/cm^2^)	1.226 (0.013)*	1.168 (0.013)	0.005	0.018–0.100
_L_BMD (g/cm^2^)	1.285 (0.099)*	1.235 (0.121)	0.015	0.010–0.091
CV (%)	7.71	9.80	N/A	N/A
Adj _L_BMD (g/cm^2^)	1.316 (0.015)*	1.205 (0.014)	< 0.001	0.067–0.155
_LS_BMD (g/cm^2^)	1.127 (0.149)	1.175 (0.178)	0.110	− 0.109 to 0.111
CV (%)	13.19	15.16	N/A	N/A
Adj _LS_BMD (g/cm^2^)	1.154 (0.023)	1.149 (0.023)	0.893	− 0.065 to 0.075
T-score	1.14 (1.05)	0.99 (1.28)	0.478	− 0.273 to 0.579
Adj T-score	1.42 (0.16)*	0.72 (0.16)	0.005	0.222–1.182
Z-score	1.05 (0.90)	1.02 (1.15)	0.847	− 0.340 to 0.413
Adj Z-score	1.33 (0.14)*	0.75 (0.14)	0.008	0.155–0.989
_T_BMD range	0.350 (1.010–1.360)	0.448 (0.991–1.439)	N/A	N/A
_L_BMD range	0.468 (1.029–1.497)	0.521 (0.993–1.513)	N/A	N/A
_LS_BMD range	0.760 (0.710–1.460)	0.700 (0.870–1.570)	N/A	N/A
T-score range	4.10 (− 1.20–2.90)	5.30 (− 1.50–3.80)	N/A	N/A
Z-score range	3.70 (− 0.90–2.80)	4.30 (− 0.80–3.50)	N/A	N/A

Data are mean (SD) except adjusted values that are mean (SE), range variables that are mean (minimum–maximum), and coefficients of variation (CV) that are percentages

_*T*_*BMD* total bone mineral density, _*L*_*BMD* leg bone mineral density, _*LS*_*BMD* lumbar spine bone mineral density, *Adj* adjusted

*Indicates difference from non-athletes

Specifically, _LS_BMD was 9% lower in male runners than non-athletes (*P* = 0.004) but there were no differences in _T_BMD (*P* = 0.176), _L_BMD (*P* = 0.963), T-score (*P* = 0.141) or Z-score (*P* = 0.092) between these two groups (Table 2). Body-mass adjusted _T_BMD and _L_BMD were 4% (*P* = 0.036) and 6% (*P* < 0.001) higher, in male runners than non-athletes but there were no differences in _LS_BMD (*P* = 0.345), T-score (*P* = 0.091) or Z-score (*P* = 0.106) between the two groups (Table 2).

_L_BMD was 4% higher in female runners than non-athletes (*P* = 0.015), but there were no differences in _T_BMD (*P* = 0.508), _LS_BMD (*P* = 0.110), T-score (*P* = 0.478) or Z-score (*P* = 0.847) between these two groups (Table 3). Body mass-adjusted _T_BMD was 5% (*P* = 0.005) and _L_BMD 9% (*P* < 0.001) higher in female runners compared to non-athletes. Body mass-adjusted T-scores (*P* = 0.005) and Z-scores (*P* = 0.008) were also higher in runners compared to non-athletes but no differences in body mass-adjusted _LS_BMD were observed between the two groups (*P* = 0.893; Table 3). No differences in _T_BMD (*P* = 0.293), _L_BMD (*P* = 0.528), _LS_BMD (*P* = 0.677), T-score (*P* = 0.295) or Z-score (*P* = 0.740) were observed between amenorrheic and eumenorrheic runners (Table 1 in supplementary material).

**Fig. 1:**
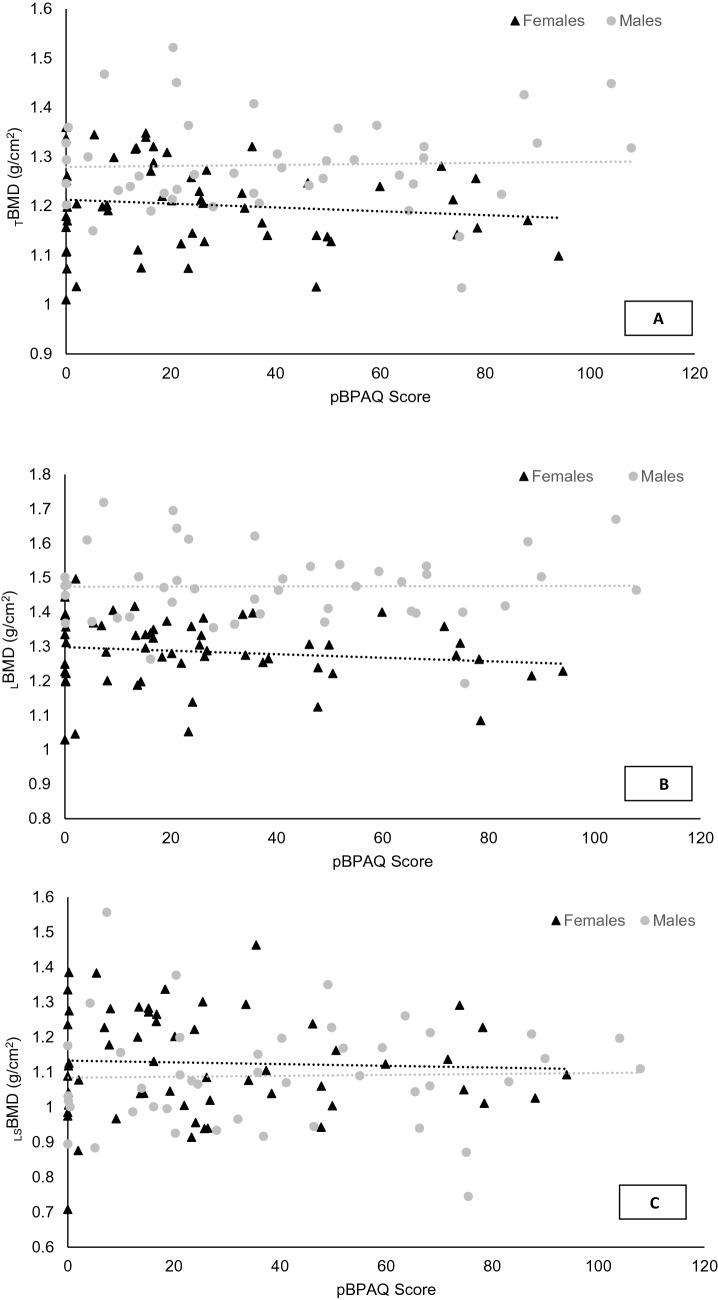
**A** Total bone mineral density (_T_BMD); **B** leg bone mineral density (_L_BMD); and **C** lumbar spine bone mineral density (_LS_BMD) in male and female high-level endurance runners in relation to their calculated past bone-specific physical activity questionnaire (pBPAQ) score

The calculated pBPAQ score (assessing the type of and number of sports completed in childhood) did not predict _T_BMD, _L_BMD or _LS_BMD in adulthood for males (*P* = 0.832, *P* = 0.962, *P* = 0.864; Fig. 1; Supplementary Material Fig. 1) or females (*P* = 0.398, *P* = 0.324, *P* = 0.781; Fig. 1; Supplementary Material Fig. 2).

## Discussion

This investigation is the first to compare bone phenotypes (_T_BMD, _L_BMD, _LS_BMD, T-score and Z-score) in high-level endurance runners with a non-athlete group, whilst also assess the impact of childhood sporting activity and menstrual status on BMD within high-level endurance runners.

Higher _L_BMD but not _T_BMD or _LS_BMD was shown in female runners compared to non-athletes. Specifically, _L_BMD was 0.050 g/cm^2^ higher in runners than non-athletes, highlighting the potential effects of site-specific mechanical loading on the lower extremity in endurance runners, which is congruent with some previous research (Brahm et al. 1997; Duncan et al. 2002; Nevill et al. 2003; Scofield and Hecht 2012). Mechanical loading initiates a response in molecular pathways mediating mechanical signalling in bone (e.g. nuclear factor k–b/nuclear factor k–b ligand/osteoprotegerin (RANK/RANKL/OPG), Wnt signalling and purinergic signalling pathways), influencing bone formation and resorption (Nakashima et al. 2011). Nevill et al. (2003) reported higher BMD in the legs of female endurance runners in comparison to upper body sites. They concluded that site-specific loading may enhance lower body BMD (via a positive osteogenic effect) at the expense of bone mass of the upper body sites, which may explain why we showed no difference in _T_BMD or _LS_BMD between female runners and non-athletes.

In one of the only other studies to investigate BMD in UK female endurance runners competing at a high-level, Pollock et al. (2010) demonstrated low _T_BMD (Z-score of − 1.0 to − 2.0) in two (4.9%) of the runners only but a median total-body Z-score of approximately 0.1 for their entire cohort. In the current study, the lowest observed total-body Z-score was − 0.9 in the female runners, with a median Z-score of 1.1 for the female runner group, suggesting higher _T_BMD in the runners we investigated. Sixty-six percent of the runners within this investigation had achieved the PB cutoff criteria for the marathon and at least one other running distance whilst differences in the mean age of the participants between the two studies existed (22 ± 6 vs 34 ± 12 years). Consequently, the impact of age as well as potential differences in the proportion of runners competing across the different running distances (> 800 m) between the two studies and the associated variation in body mass, running training volume and/or strength and resistance-based training practices could have contributed to the differences in findings.

Greater running distance per week has been negatively correlated with BMD (Burrows et al. 2003; Hind et al. 2006), but higher level runners (such as those participating in our investigation) are more likely to undertake resistance/strength training (Blagrove et al. 2017), which may apply greater amounts of high and multi-directional force to the bone, consequentially benefiting BMD (Nevill et al. 2003). Runners who complete higher volumes of resistance training have higher _LS_BMD that those who may complete lower volumes (Gordon and Nelson 2003), which may explain why we showed no difference in _LS_BMD between female runners and non-athletes.

Lower _LS_BMD but no differences in _T_BMD or _L_BMD were present in male runners. It is surprising to report no difference in _L_BMD between the male runners and non-athletes, given the differences observed in the female runners within this study. Previous investigation has also reported _L_BMD can be up to 14% higher in runners compared to non-athletes (Stewart and Hannan 2000; Kemmler et al. 2006). Kemmler et al. (2006), however, only investigated 20 high-level runners whilst Stewart and Hannan (2000) investigated bone phenotypes in runners with a range of ability, from club to international level, with specific racing distances not stated. Lower _LS_BMD in runners compared to non-athletes observed in our investigation is, however, comparable with other research. Lower vertebral (but not tibial or radial) BMD has been shown in male endurance runners completing 92.2 ± 6.3 km per week (Bilanin et al. 1989), whilst Hind et al. (2006) and Fredericson et al. (2007) reported low _LS_BMD in comparison to a reference population in male endurance runners. Endurance runners tend to have lower body mass than non-athletes (as shown in this study; Tables 2 and 3) and thus, if all else is equal, lower load will be exerted on these anatomical sites than in non-athletes. In addition, as the lumbar spine is considered a site of relatively less loading during endurance running (Pollock et al. 2010), less mechanical loading will occur here compared to the lower extremity, which might explain why BMD was lower at this site in runners compared to non-athletes, despite no difference in _L_BMD and _T_BMD (Cappozzo 1983; Pollock et al. 2010). However, other factors such as genetic predisposition, hormones and nutritional intake may also influence BMD in endurance runners.

Male runners may be at risk of relative energy deficiency in sport (RED-S), as highlighted in the recent IOC consensus statement (Mountjoy et al. 2018). Low energy availability induced by insufficient dietary intake and/or excessive energy expenditure may increase bone resorption and negatively impact bone metabolic markers, resulting in decreased bone formation, lower bone mass and altered structure (Papageorgiou et al. 2018). The benefits of mechanical loading on BMD, could, therefore, be lost, or reduced, by energy deficiency. Although difficult to assess directly from circulating bone (re)modelling markers, the balance of bone metabolism following repeated training in male endurance runners does not appear to be affected unless an energy deficiency is present, resulting in suppression of bone formation (Papageorgiou et al. 2018). A greater magnitude of loading at the lower extremity and the associated mechanical impact from running may protect against the potentially negative effect of reduced energy availability on BMD and consequently explain why lower _LS_BMD but similar _T_BMD and _L_BMD were shown compared to non-athletes. Energy availability was not measured in this investigation due to the difficulty in accurately measuring this complex phenomenon in such a large sample, so this surmised influence is based upon previous literature. The current methods available to assess energy availability are not without difficultly and consequently, it remains extremely problematic to identify “true” energy availability (Logue et al. 2020).

It is interesting that lower _LS_BMD was only evident in male, and not female, runners in comparison to their non-athlete counterparts. Higher oestrogen may preserve _LS_BMD in female runners. Indeed, studies reporting lower _LS_BMD in female endurance runners versus non-runners have primarily been in those who may have low energy availability and/or menstrual irregularities (Barrack et al. 2008; Scofield and Hecht 2012). However, we observed no difference in BMD at any site between amenorrheic and eumenorrheic runners (data appeared slightly lower in amenorrheic runners but did not approach statistical significance), suggesting that menstrual status did not affect BMD in our cohort. We assessed potential amenorrhoea and the number of sports completed in childhood via self-report questionnaire. Whilst measurement error exists (Small et al. 2007; Prince et al. 2008), questionnaires are inexpensive and easy to implement in larger cohorts, and widely used (Hoch et al. 2009; Farr et al. 2011; Martin et al. 2017). Other parameters that may influence bone phenotypes, such as smoking history and alcohol intake were not assessed as part of this investigation. Obtaining such information via self-report may not be particularly representative of the truth (Gorber et al. 2009), which in turn impacts the ability to assess or account for these parameters appropriately.

Physical activity during childhood is a key period for bone accretion (Weaver et al. 2016). Therefore, a limited range of physical activities during childhood could have negative implications for adult BMD. Herein, however, we identified no association between pBPAQ score (the type of sport and the number of sports completed in childhood) and any bone phenotype. Consequently, our findings suggest an appropriate volume of physical activity is completed (in childhood) to provide sufficient loading and associated mechanosensory benefit to elevate BMD in most runners.

Of note, we observed higher body-mass adjusted bone phenotypes for both male (_T_BMD and _L_BMD) and female (_T_BMD, _L_BMD, T-score and Z-score) runners in comparison to their non-athlete counterparts. Greater body mass has been shown to positively influence BMD, likely due to the increased load experienced by the bone (Felson et al. 1993). However, when body mass is accounted for, runners demonstrated higher relative BMD, possibly as a result of completing larger volumes of physical activity and benefitting from the associated mechanostransductive effect, than their non-athlete counterparts. The impact of body mass on BMD, however, is influenced by both muscle and fat tissue mass differently as well as the complex relationships between these body composition components and mechanical factors (Bierhals et al. 2019).

The large variance in _T_BMD, _L_BMD and _LS_BMD in both non-athletes and endurance runners is notable. This cannot be attributed solely to age- or physical activity-associated effects on BMD and indicates that other factors such as genetic variation also influence BMD. Heritability of BMD is estimated at 50–85% (Ralston and Uitterlinden 2010) and numerous genes may play a role (Hsu and Kiel 2012; Golchin et al. 2016).

## Conclusion

The findings of this study in high-level endurance runners suggest some may have higher, or similar, BMD at sites experiencing higher mechanical loading but lower BMD at less-loaded sites when compared to non-athletes. These results are consistent with previous research on smaller cohorts (Kemmler et al. 2006), younger populations (Duncan et al. 2002) and in athletes of undefined/lower ability (Hind et al. 2006) but are the first to demonstrate such results in a larger cohort of high-level Caucasian endurance runners, selected based upon PB. Female runners had higher _L_BMD but not _T_BMD or _LS_BMD than female non-athletes, whilst male runners possessed but similar _T_BMD and _L_BMD compared to non-athletes. Male runners, however, also displayed lower BMD at the lumbar spine in comparison with non-athletes, which, hypothesised, may be due to the presence of reduced energy availability. Menstruation status and the number of sports completed in childhood did not appear to influence bone phenotypes, although large variability in BMD was observed in both the male and female runners and non-athletes, suggesting that other factors such as genetic variation, diet and types of loading influence BMD across the adult lifespan.

## Supplementary Information

Below is the link to the electronic supplementary material.

Supplementary file1 (DOCX 14 KB)

Supplementary file2 (DOCX 17 KB)

Supplementary file3 (DOCX 35 KB)

## Data Availability

The data sets generated during and/or analysed during the current study are available from the corresponding author on reasonable request.

## Abbreviations

BPAQ: Bone Physical Activity Questionnaire
BMD: Bone mineral density
DXA: Dual-energy X-ray absorptiometry
MANOVA: Multiple analysis of variance
MANCOVA: Multiple analysis of covariance
RED-S: Relative energy deficiency in sport
