# Correction to: Bone mineral density in high-level endurance runners: part A—site-specific characteristics

**DOI:** 10.1007/s00421-021-04818-x

**Published:** 2021-10-19

**Authors:** A. J. Herbert, A. G. Williams, S. J. Lockey, R. M. Erskine, C. Sale, P. J. Hennis, S. H. Day, G. K. Stebbings

**Affiliations:** 1 School of Health Sciences, Birmingham City University, Birmingham, UK; 2 Sports Genomics Laboratory, Department of Sport and Exercise Sciences, Manchester Metropolitan University, Manchester, UK; 3 Faculty of Health, Education, Medicine and Social Care, Anglia Ruskin University, Chelmsford, UK; 4 School of Sport and Exercise Science, Liverpool John Moores University, Liverpool, UK; 5 Musculoskeletal Physiology Research Group, Sport, Health and Performance Enhancement Research Centre, School of Science and Technology, Nottingham Trent University, Nottingham, UK; 6 School of Medicine and Clinical Practice, University of Wolverhampton, Wolverhampton, UK; 7 Institute of Sport, Exercise and Health, University College London, London, UK

## Correction to: European Journal of Applied Physiology 10.1007/s00421-021-04793-3

The original version of this article unfortunately contained a mistake. Figure 1C was missing.

The corrected Fig. 1 should have appeared as shown in the following page.

**Fig. 1:**
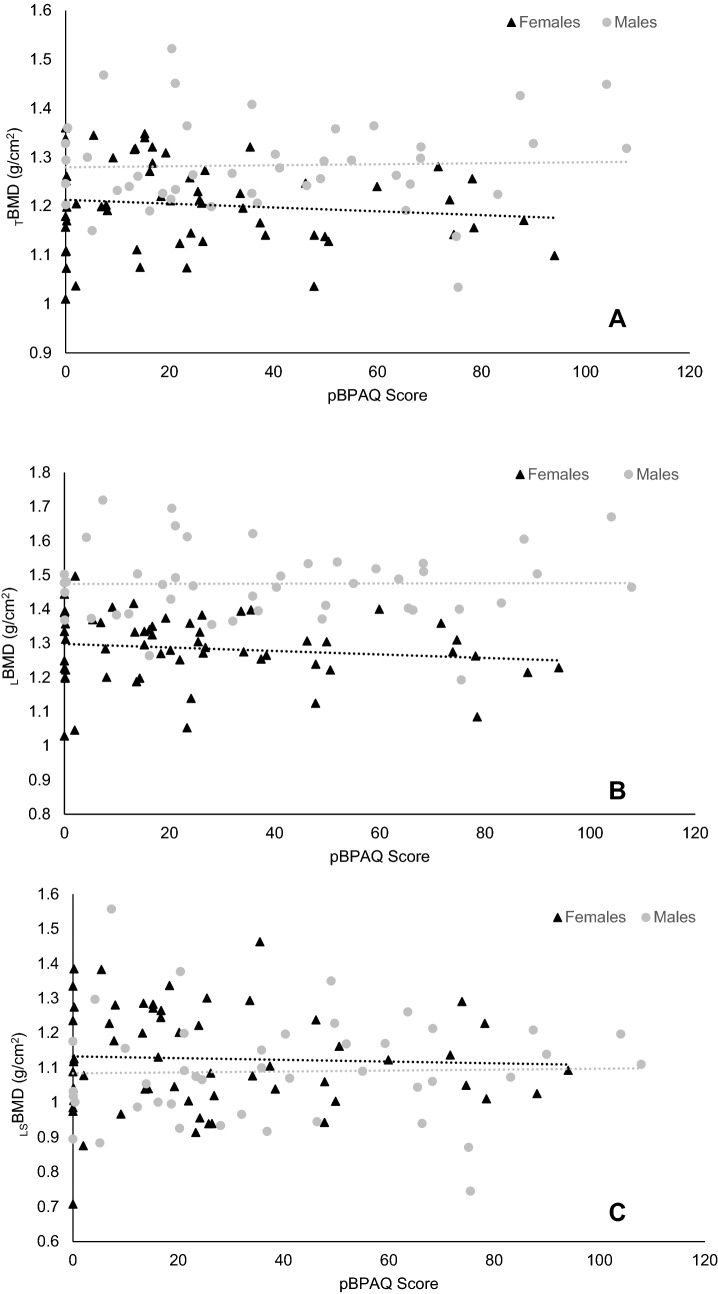
**A** Total bone mineral density (_T_BMD); **B** leg bone mineral density (_L_BMD); and **C** lumbar spine bone mineral density (_LS_BMD) in male and female high-level endurance runners in relation to their calculated past bone-specific physical activity questionnaire (pBPAQ) score

The original article has been corrected.

