# Supplementary material for: Bone mineral density in high-level endurance runners: part A—site-specific characteristics
Source: Eur J Appl Physiol. 2021 Sep 12;121(12):3437–45. doi: 10.1007/s00421-021-04793-3 (PMC8571133; doi:10.1007/s00421-021-04793-3)
Supplement: Supplementary file 1 — Supplementary file1 (DOCX 14 KB) [file 421_2021_4793_MOESM1_ESM.docx]

**Table 1:** Anthropometric characteristics and unadjusted bone phenotype data in eumenorrheic and amenorrheic high-level endurance runners.

|  | Eumenorrheic (n=40) | Amenorrheic (n=18) |
| --- | --- | --- |
| Age (years) | 37 (11) | 27 (12) |
| Height (m) | 1.64 (0.06) | 1.66 (0.06) |
| Mass (kg) | 52.9 (4.9) | 53.0 (6.0) |
| _T_BMD (g/cm^2^) | 1.211 (0.089) | 1.185 (0.086) |
| _L_BMD (g/cm^2^) | 1.290 (0.094) | 1.272 (0.111) |
| _LS_BMD (g/cm^2^) | 1.132 (0.146) | 1.114 (0.158) |
| T-score | 1.25 (1.04) | 0.94 (1.02) |
| Z-score | 1.08 (0.91) | 0.99 (0.89) |

Data are presented as mean (SD). _T_BMD = Total Bone Mineral Density; _L_BMD = Leg Bone Mineral Density; _LS_BMD = Lumbar Spine Bone Mineral Density.
