# Supplementary material for: Bone mineral density in high-level endurance runners: part A—site-specific characteristics
Source: Eur J Appl Physiol. 2021 Sep 12;121(12):3437–45. doi: 10.1007/s00421-021-04793-3 (PMC8571133; doi:10.1007/s00421-021-04793-3)
Supplement: Supplementary file 3 — Supplementary file3 (DOCX 35 KB) [file 421_2021_4793_MOESM3_ESM.docx]

**Fig. 2:** Total bone mineral density (_T_BMD), leg bone mineral density (_L_BMD) and lumbar spine bone mineral density (_LS_BMD) in female runners depending upon the number of sports participated in within childhood.
